# Zhenwu decoction ameliorates adriamycin-induced nephrotic syndrome in rats via dual modulation of AVP-V2R-AQP2 and RAAS-MR-AQP3 pathways

**DOI:** 10.1080/0886022X.2025.2599570

**Published:** 2025-12-16

**Authors:** Mengmeng Xu, Min Du, Wanying Qu, Yanjun Wei, Hui Wang, Xin Yang

**Affiliations:** ^a^ School of Basic Medicine, Guizhou University of Traditional Chinese Medicine, Guiyang, China; ^b^ Department of Gastroenterology, First Affiliated Hospital of Guizhou, University of Traditional Chinese Medicine, Guiyang, China; ^c^ School of Health Preservation, Guizhou University of Traditional Chinese Medicine, Guiyang, China

**Keywords:** Zhenwu decoction, nephrotic syndrome, traditional chinese medicine (TCM), Renin-Angiotensin-Aldosterone system (RAAS), renal fibrosis, doxorubicin nephropathy

## Abstract

The core bioactive components of Zhenwu Decoction (ZWD)—utilized for managing kidney diseases—and the associated anti-nephrotic syndrome (NS) edema mechanism remain incompletely characterized. We assessed the potential mechanism of ZWD against adriamycin (ADR)-induced NS. Sprague–Dawley rats (*n* = 6/group) received intravenous adriamycin (6 mg/kg) to establish NS models and were administered ZWD (4.2, 8.4, 16.8 g/kg/day) and prednisone (5 mg/kg/day) for 4 weeks. Renal function was assessed using the serum biomarkers: blood urea nitrogen (BUN), serum total protein (TP), creatinine (Scr), albumin (ALB), triglycerides (TG), total cholesterol (TC), high-density lipoprotein cholesterol (HDL-C), and low-density lipoprotein cholesterol (LDL-C). H&E and Masson’s trichrome staining were used to assess the pathological alterations and renal fibrosis status. Enzyme-linked immunosorbent assay was used to detect the serum levels of arginine vasopressin (AVP), renin (Ren), angiotensin II (AngII), and aldosterone (ALD). Immunohistochemistry, Western blot, and RT-qPCR were performed to examine the levels of relevant intracellular renal signaling molecules. ZWD treatment significantly reduced proteinuria by 58% in NS rats, along with marked improvements in renal pathology and fibrosis. ZWD also ameliorated serum levels of TP and LDL-C, and lowered the concentrations of AVP, Renin, AngII, and ALD. The expression of type 2 vasopressin receptor (V2R), mineralocorticoid receptor (MR), aquaporin 1 (AQP1), AQP2, and AQP3 was remarkably decreased at the protein and mRNA levels. Three components (benzoylpaeoniflorin, 6-gingerol, and paeoniflorin) of ZWD exhibited synergistic effects on AQP1, AQP2, AQP3, MR, and V2R. Our findings suggest that ZWD alleviates NS through renal repair and dual suppression of the AVP-V2R-AQP2 and RAAS-MR-AQP3 pathways.

## Graphical Abstract

**Figure:**
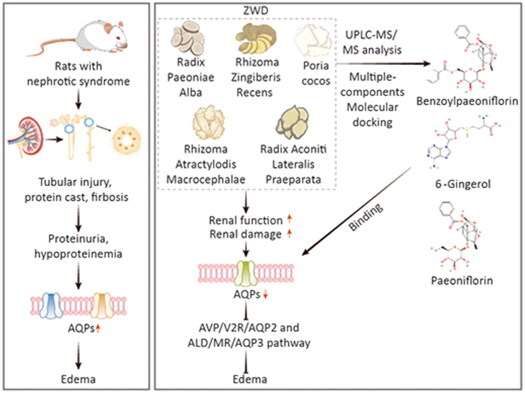

## 1. Introduction

Nephrotic syndrome (NS) is a disease of the urinary system that causes deleterious effects and increases the risk of end-stage renal failure [1]. Edema, a typical clinical symptom of NS, has been proved to be related to the abnormally high expression of water channel aquaporin 2 (AQP2) in the kidneys of both humans and rats [2,3]. AQP2 plays an essential role in facilitating water reabsorption in the collecting ducts (CDs) through a type 2 vasopressin receptor (V2R)-mediated system [4]. AQP3 is expressed on the basolateral plasma membrane of the same principal cells as AQP2 and represents an exit pathway for water reabsorption *via* AQP2 [5], which can be regulated by the hormone, aldosterone (ALD) [6]. In addition, upregulation of the renin-angiotensin-aldosterone system (RAAS) has been identified as a major mediator of renal injuries [7].

Zhenwu decoction (ZWD), a classical Chinese herbal formula, has been extensively used for centuries in Chinese clinical practice for managing kidney diseases [8]. ZWD comprises *Aconitum carmichaelii Debeaux* (Fuzi), *Poria cocos* (Fuling), *Atractylodes macrocephala Koidz* (Baizhu), *Paeonia lactiflora Pall* (Baishao), and *Zingiber oficinale Roscoe* (Shengjiang). Modern pharmacological studies have demonstrated that the clinical efficacy of ZWD against NS through its triple action: renoprotective, diuretic, and anti-inflammatory profile [9–12]. The consistency of the chemical constituents of ZWD before and after compatibility testing has been investigated using metabolomics [13]. *P. cocos*, the most important diuretic component of ZWD, suppressed renal AQP2 expression in NS rats [14,15]. However, the core bioactive components of ZWD and the mechanism associated with its anti-NS edema activity remain incompletely characterized.

Accordingly, to characterize the bioactive components of ZWD, we employed ultra-high-performance liquid chromatography-tandem mass spectrometry (UPLC-MS/MS), followed by molecular docking to identify its primary active constituents against NS edema. Furthermore, using an adriamycin (ADR, also called doxorubicin)-induced NS murine model, we investigated the changes in the arginine vasopressin (AVP)-V2R-AQP2 and RAAS-mineralocorticoid receptor (MR)-AQP3 pathways to elucidate the regulatory mechanism underlying the diuretic effect of ZWD. Our findings not only clarify the pharmacodynamic material basis of ZWD but also elucidate its mechanism of action, thereby providing a foundation for its clinical application and guiding further experimental research.

## 2. Materials and methods

### 2.1. Preparation of ZWD

The ZWD used in this study comprised five components: *Aconitum carmichaelii Debeaux* (Fuzi) 9 g, *Poria cocos* (Fuling) 9 g, *Atractylodes macrocephala* Koidz (Baizhu) 6 g, *Paeonia lactiflora* Pall (Baishao) 9 g, and *Zingiber oficinale* Roscoe (Shengjiang) 9 g. Baizhu was purchased from Guangjitang Pharmacy (Yulin, China) and all other components were purchased from Beijing Tongrentang Pharmacy (Beijing, China). The botanical drug was identified by Professor Wang Hui, and the voucher specimen has been deposited in the Herbarium of Guizhou University of Traditional Chinese Medicine under the accession number TFL-2022-06. All the raw materials of the ZWD prescription were boiled for 2 h after being steeped in eight times distilled water for 1 h. The ZWD extract was filtered and concentrated using a rotary evaporator. The aqueous extract (1.68 g raw material/ml) was obtained as previously described and stored at 4 °C [16].

### 2.2. Ultra-high performance liquid chromatography (UPLC) analysis

The chemical constituents of ZWD were analyzed using a Sciex ExionLC^™^AD Ultra-High Performance Liquid Chromatography Tandem Mass Spectrometry (UPLC-MS/MS) system with an Agilent SB-C18 analytical column (100 × 2.1 mm, 1.8 μm), as previously described [12]. For sample preparation, a 200 μL aliquot of the prepared ZWD aqueous extract was mixed with 200 μL of internal standard extraction solution (prepared in 70% methanol). After vortexing for 15 min, the mixture was centrifuged at 12,000 rpm for 3 min at 4 °C. The supernatant (1 mL) was filtered through a 0.22 mm microporous membrane before UPLC–MS/MS analysis.

### 2.3. Animals

Sixty specific pathogen–free (SPF) male Sprague–Dawley rats (180–220 g) were purchased from Tianqin Biotechnology (Changsha, Hunan Province, China), with a certificate number of [SCXK (Xiang) 2014-0011]. The rats were maintained under SPF conditions at 20–23 °C, 50–60% humidity, a 12-h dark/light cycles, and with food and water supplied ad libitum. All procedures were performed according to the requirements of the Animal Care and Welfare Committee of Guizhou University of Traditional Chinese Medicine. All animals successfully survived the entire modeling process.

### 2.4. Experimental design

After 1 week of acclimatization, the NS model was prepared *via* a single tail vein injection of ADR (6 mg/kg, prepared in 0.9% NaCl solution) to accurately mimic the clinical features of NS in humans, as previously described [11,17]. The rats in the control group were injected with the same dose of normal saline. Furthermore, we performed 24 h proteinuria tests to confirm model establishment on days 7 and 14 post-ADR injection. Based on urinary protein levels, 36 SD rats exhibiting the most characteristic proteinuria were randomly divided into 6 groups (*n* = 6 in each group): control, ADR, ZWD (4.2, 8.4, 16.8 g/kg), and prednisone (5 mg/kg). ZWD at 4.2 g/kg was selected based on the body surface area conversion between humans and rats [11], while 8.4 g/kg and 16.8 g/kg were selected as previously described [12,16]. The rats in the control and ADR groups were administered normal saline, whereas those in the ZWD and prednisone groups were administered *via* oral gavage once daily for 4 weeks.

During the 4-week administration, 24 h urine samples were collected using commercial metabolic cages on days 0, 7, 14, 21, and 28. The animals were fasted with free access to water, and urine volume was recorded at the time of collection. On day 28, all animals were anesthetized with isoflurane (5% for induction, 2% for maintenance) for terminal blood collection from the abdominal aorta. Following blood collection, euthanasia was ensured by cervical dislocation while the animals remained under deep anesthesia. Serum was obtained by centrifugation of the blood samples and stored at −80 °C. Kidneys were removed and fixed in 4% paraformaldehyde or stored at −80 °C for subsequent testing. Body weight was recorded once per week.

### 2.5. Kidney index

All the rats were weighed after anesthesia, and kidney tissues were removed and weighed immediately after anesthetization and euthanasia. The kidney index was calculated as the ratio of the absolute kidney weight to the body weight.

### 2.6. Blood and uric index analysis

Blood samples were analyzed using an automatic biochemical analyzer (Chemray240). Appropriate assay kits were used to measure the serum total protein (TP; Cat. No. C007-d), creatinine (Scr; Cat. No. C074-d), albumin (ALB; Cat. No. C008-d), triglycerides (TG; Cat. No.C019-a), total cholesterol (TC; Cat. No. C048-a), blood urea nitrogen (BUN; Cat. No.C010-a), high-density lipoprotein cholesterol (HDL-C; Cat. No.C046-a), and low-density lipoprotein cholesterol (LDL-C; Cat. No.C047-a). Serum AVP (Cat. No.H396-1), renin (Ren; Cat. No. MM-0343R2), Angiotensin II (AngII; Cat. No. MM-021R2), and ALD (Cat. No. H188) levels were measured using ELISA kits. Urine protein and urine sodium levels were measured using assay kits (Cat. No. C035-2; Cat. No.C002-1).

### 2.7. Histopathology assay

Kidney tissues were fixed in 4% paraformaldehyde for 24 h, embedded in paraffin, and cut into 4-μm thick sections. Slices were deparaffinized in xylene and rehydrated *via* an alcohol series before treatment with hematoxylin and 1% eosin for Hematoxylin and eosin (H&E) staining. Images were captured using an optical microscope (Nikon). The severity of renal tissue injury was evaluated using a five–level system (0–4): normal (0), 0–25% (1), 25–50% (2), 50–75% (3), and >75% (4) [18]. Masson’s trichrome staining of the kidneys was performed according to the manufacturer’s protocol of (BA4079, BASO, China). Myofibers and collagen fibers are stained red and blue, respectively. The degree of fibrosis was quantified as the ratio of the Masson-positive area to the total field area using digital pathology image-analysis software (Aipathwell; Servicebio, Beijing, China).

### 2.8. Immunohistochemistry (IHC)

After dewaxing and antigen retrieval, the renal slices were incubated in 3% hydrogen peroxide solution for 15 min to eliminate endogenous peroxidase. The slices were blocked with 10% goat serum at room temperature and incubated with the primary antibodies against AQP1 (diluted 1:100, NB600-749, NOVUS, USA), AQP2 (diluted 1:100, DF7560, Affinity, USA), and AQP3 (diluted 1:1000, BS-1253R, BIOSS, China) were added and incubated at 4 °C overnight. The next day, the renal slices were incubated with secondary antibodies at room temperature for 1h. The immunoreactive sites were subsequently stained using the 3,3′- diaminobenzidine (DAB substrate kit; Servicebio), and visualized using a Nikon Fi3 biological microscope. Four fields of view per section were randomly selected and analyzed quantitatively using Image-Pro Plus 6.0 software (NIH, Bethesda, USA).

### 2.9. Western blot (WB) analysis

The kidneys were lysed in buffer containing 1 mM phenylmethylsulfonyl fluoride mixed with a phosphorylated protease inhibitor cocktail (MB12707, Meilunbio, China), and the protein concentration was determined using a BCA protein assay. The proteins were loaded onto PVDF membranes blocked with 5% nonfat milk at room temperature for 2 h and incubated with the primary antibodies, horseradish peroxidase–conjugated secondary antibodies, and enhanced chemiluminescence (ECL) reagent (KF8003, Affinity). Protein levels were analyzed using Image-Pro Plus 6.0 software (NIH, Bethesda, USA), with β-actin as the internal control.

The primary antibodies used were against MR (diluted 1:500, BS-1850R, BIOSS), V2R (diluted 1:1000, BS-10014R, BIOSS), AQP1 (diluted 1:1000, AF5231, Affinity), AQP2 (diluted 1:2000, DF7560, Affinity), AQP3 (diluted 1:2000, BS-1253R, BIOSS), and β-actin (diluted 1:30000, 66009-1-Ig, Proteintech, USA).

### 2.10. Real time quantitative PCR (RT-qPCR)

RT-qPCR was performed to determine the mRNA levels of AQP1, AQP2, AQP3, V2R, and MR. Total RNA from the kidney tissues were extracted using TRIzol reagent (15596-026, Ambion, USA) and reverse-transcribed into cDNA. The RT-qPCR conditions were as follows: pre-denaturation for 10 min at 95 °C, 40 cycles of 15 s at 95 °C, 60 s at 60 °C, and 15 s at 95 °C. β-actin was used as internal reference, and each sample was analyzed three times. The primers used for amplifying AQP1, AQP2, AQP3,V2R, MR, and β-actin were designed using NCBI Primer, and synthesized by Huayan Biotech Co. Ltd. (Wuhan, China). The primer sequences are listed in Table 1.

**Table 1.** Primers used for RT-qPCR.

Gene	Primer	Sequence (5′–3′)
Rat β-actin	Forward	TGACGTTGACATCCGTAAAGACC
	Reverse	GTGCTAGGAGCCAGGGCAGTAA
Rat AQP1	Forward	ACCTCAACCCAGCGGTCACAC
	Reverse	GCAACGATGGCTCCCACACAC
Rat AQP2	Forward	TTCTTGGCCACGCTCCTTTTT
	Reverse	AGGTCCCCACGGATTTCTACT
Rat AQP3	Forward	GACTTTGGACCTCGCCTTTTC
	Reverse	GCCAGCTTCACATTCTCTGCC
Rat V2R	Forward	CAGGTTCTTATCTTCCGGGAG
	Reverse	TGAGCAACACAAAGGGGGGTC
Rat MR	Forward	AATAAAGCAAGAGTCAAGCAA
	Reverse	CCATAAAGGAAAAGTAGGAGC

### 2.11. Molecular docking

The chemical structures of the ZWD components were obtained from PubChem. The crystal structure of MR (PDB ID: 2AA2), V2R (PDB ID: 7R0C), AQP1 (PDB ID: 1FQY), AQP2 (PDB ID: 4NEF), and AQP3 (PDB ID:8y8o) were retrieved from the RCSB Protein Data Bank (http://www.rcsb.org). Molecular docking was performed using the Surflex-Dock module in SYBYL-X software (version 2.1.1) after processing proteins and small molecule compounds by removing water and solvent molecules and adding hydrogen atoms. Docking results were evaluated using the total score metric as the primary threshold, which comprehensively integrates the polar, hydrophobic, and solvation energy contributions, with higher scores indicating stronger binding affinities between ligands and target proteins.

### 2.12. Statistical analysis

Statistical analysis was performed using SPSS (version 26.0; IBM Corp., Armonk, NY, USA), and the data are expressed as mean ± standard deviations (SD). One-way analysis of variance (ANOVA), followed by least significant difference (LSD), was used for unpaired comparisons, and differences were considered significant at *p* < 0.05.

## 3. Results

### 3.1. UPLC–MS/MS assay of ZWD

**Figure 1.**
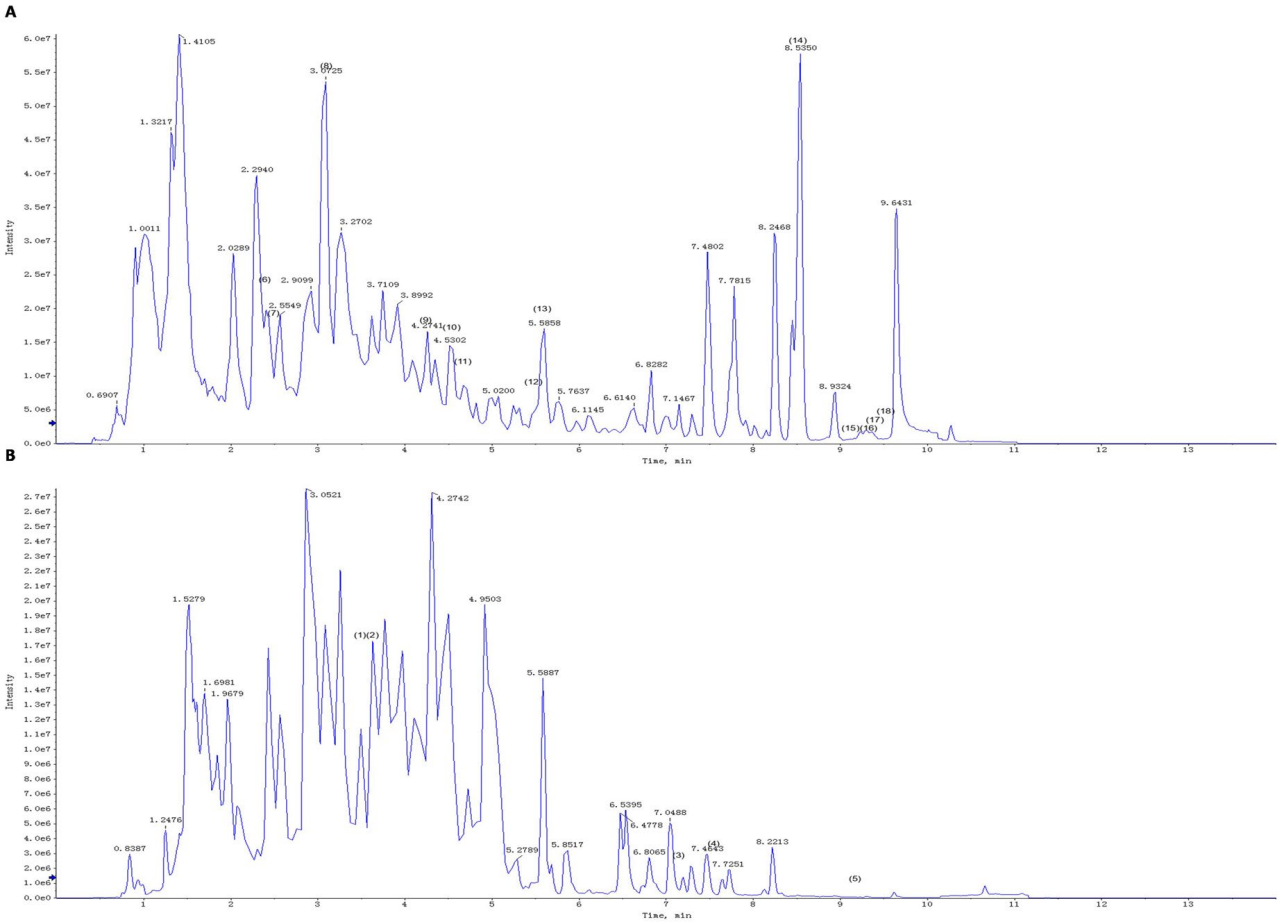
UPLC–MS/MS chromatogram of ZWD extract in (A) positive and (B) negative ion mode. UPLC–MS/MS: Ultra-High Performance Liquid Chromatography Tandem Mass Spectrometry; ZWD: Zhenwu Decoction.

The aqueous extracts of ZWD were analyzed by UPLC-MS/MS in the positive and negative ion modes (Figure 1). Eighteen bioactive compounds of ZWD have been identified: karakoline (1), (S)-norcoclaurine (2), benzoylmesaconine (3), benzoylhypaconine (4), benzoylaconine (5), paeoniflorin (6), albiflorin (7), lactiflorin (8), benzoylpaeoniflorin (9), pachymic acid (10), poricoic acid A (11), poricoic acid C (12), tumulosic acid (13), 6-gingerol (14), atractylenolide I (15), atractylenolide III (16), 3β-hydroxyatractylon (17), and catechin (18).

### 3.2. ZWD alleviates proteinuria in NS

**Figure 2.**
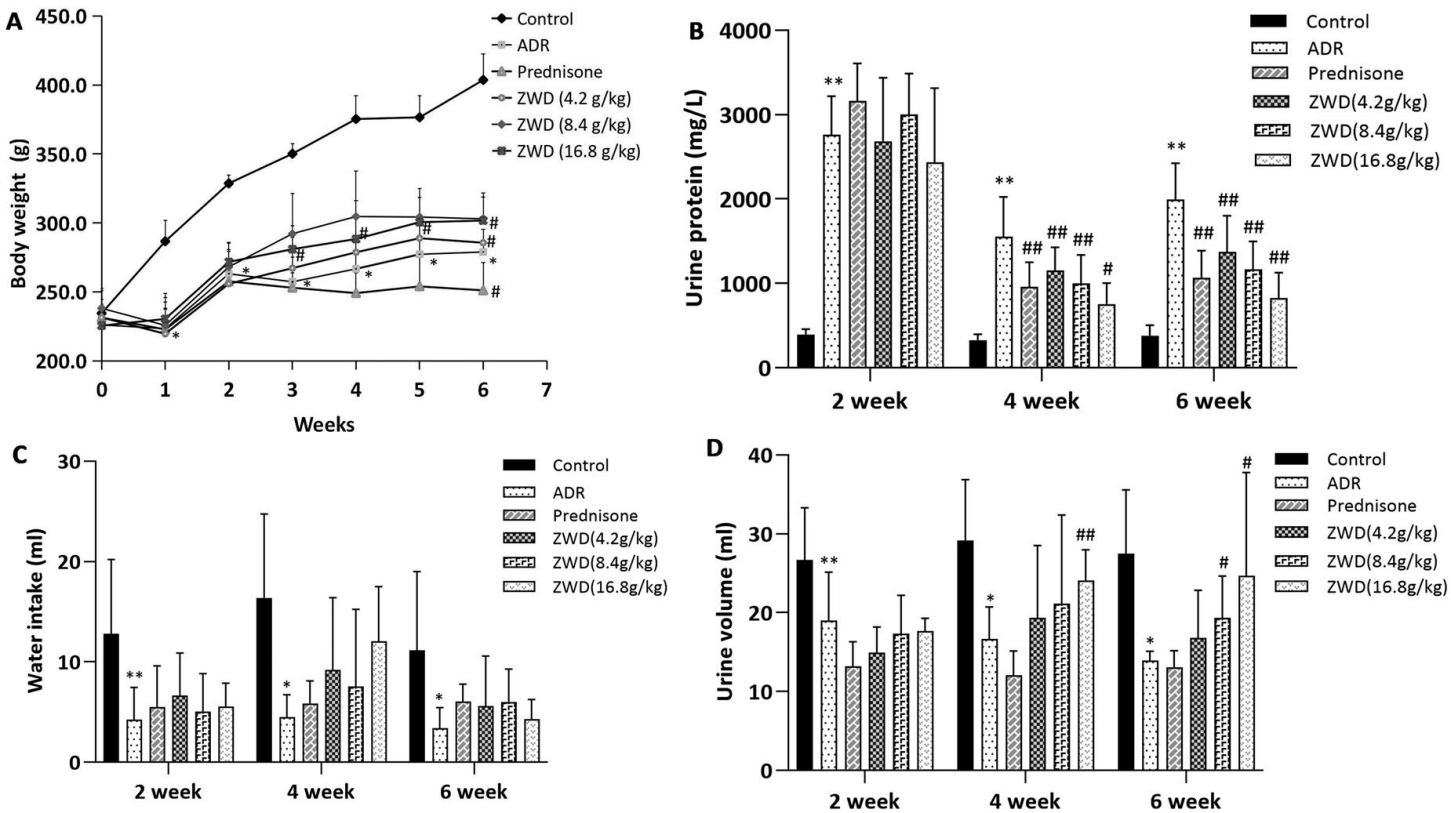
ZWD alleviates proteinuria in NS. (A) Time courses of body weight (once weekly, *n* = 6). (B) Time courses of proteinuria (twice per week; *n* = 6). (C) Time course of water intake (twice weekly, *n* = 6). (D) Time course of urine volume (twice weekly, *n* = 6). Model group vs. control group (*p < 0.05, **p < 0.01, ANOVA followed by LSD); ZWD and prednisone groups vs. model group (#*p <* 0.05, ##*p <* 0.01, ANOVA followed by LSD). ZWD: Zhenwu Decoction; LSD: least significant difference; ANOVA: analysis of variance.

Compared to the control group, urine protein levels were significantly higher (*p* < 0.05, Figure 2(A–B)), whereas body weight, water intake and urine volume were lower in the ADR group (*p* < 0.05, Figure 2(C–D)), indicating successful establishment of the NS model. ZWD at 16.8 g/kg reduced proteinuria by 58% compared to the ADR group (*p* < 0.05), and increased body weight and urine excretion in NS rats (*p* < 0.05), without a significant increase in water intake. In contrast to the limited effect of prednisone on urine output, ZWD exhibited a pronounced diuretic effect.

### 3.3. ZWD improves serum lipid in NS

**Figure 3.**
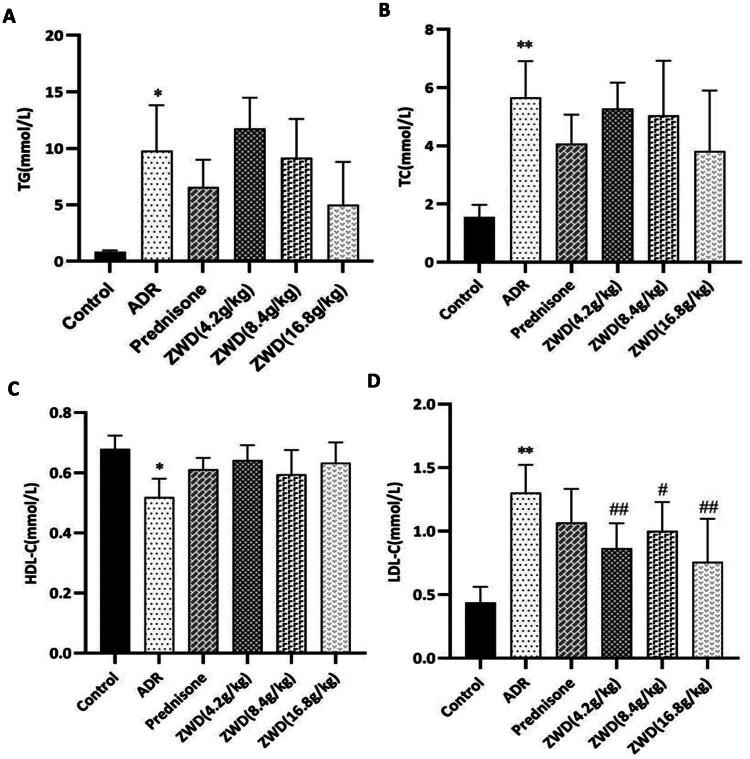
ZWD improves serum lipid levels in NS. (A) TG (*n* = 6). (B) TC (*n* = 6). (C) HDL-C (*n* = 6). (D) LDL-C (*n* = 6). Model group vs. Control group (*p < 0.05, **p < 0.01, ANOVA followed by LSD); ZWD and prednisone groups vs. model group (^#^*p <* 0.05, ^##^*p <* 0.01, ANOVA followed by LSD). ZWD: Zhenwu Decoction; LSD: least significant difference; TG: triglycerides; TC: total cholesterol; HDL-C: high-density lipoprotein cholesterol; LDL-C: low-density lipoprotein cholesterol; ANOVA: analysis of variance.

After injection with ADR for 6 weeks, serum TG, TC, and LDL-C levels increased (*p* < 0.05, Figure 3(A–C)), while HDL-C levels decreased (*p* < 0.05, Figure 3(D)). Interestingly, ZWD treatment decreased serum LDL-C levels (*p* < 0.05). Therefore, ZWD appears to improve hyperlipidemia in NS rats, whereas prednisone had little effect. Thus, these findings indicated that the therapeutic outcome of ZWD was better than that of prednisone.

### 3.4. ZWD ameliorates renal histopathology injury and fibrosis in NS

**Figure 4.**
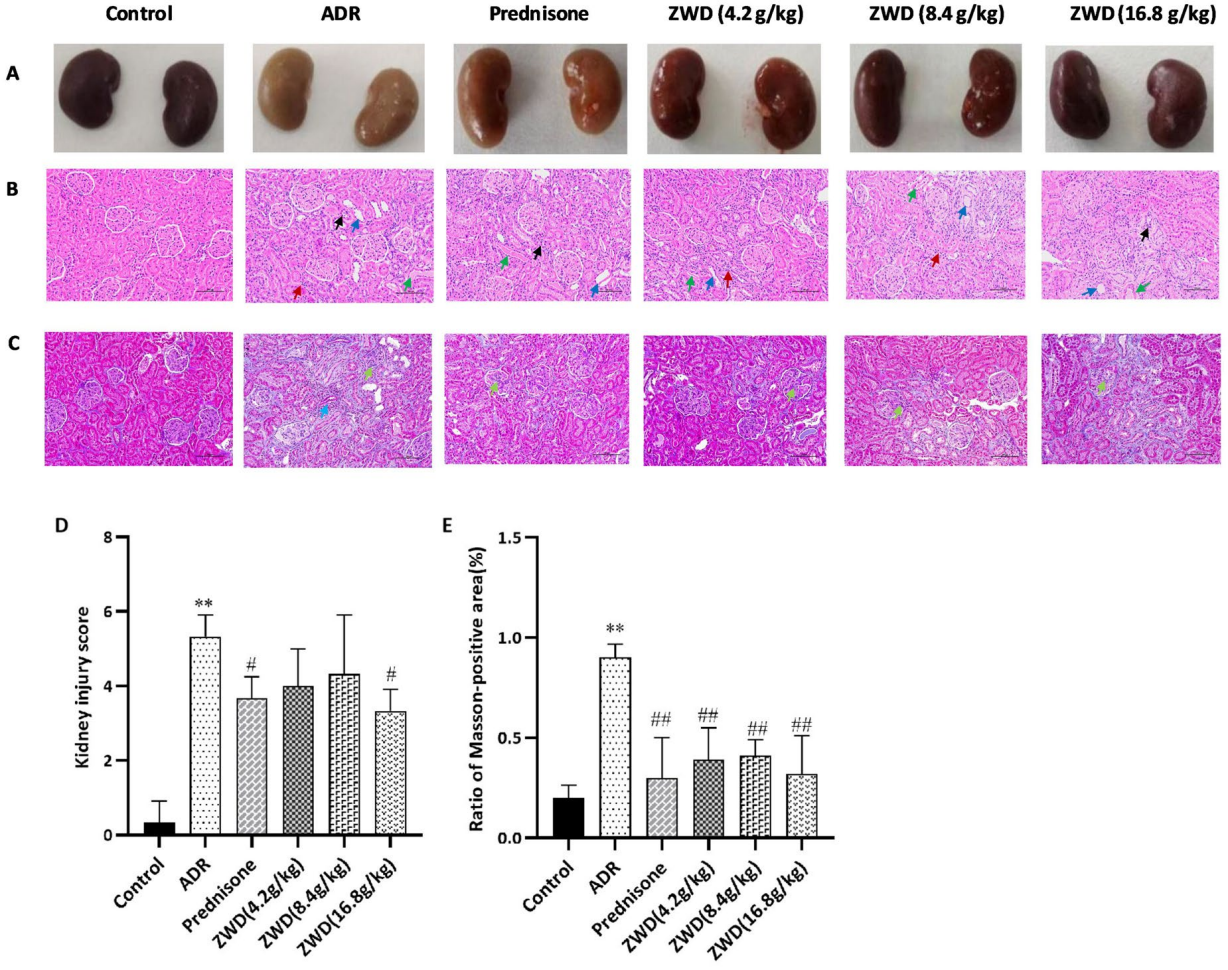
ZWD alleviates kidney structural impairment and fibrosis in NS. (A) Morphology of rat kidneys in each group. (B) H&E staining of structural impairment. (C) Masson’s trichrome staining of renal fibrosis. (D) Kidney injury score. (E) Ratio of Masson-positive area. Scale bar = 100 μm, 200× magnification. Note: In the renal pathology images, the blue arrow indicates significant dilatation of the renal tubular lumen; green arrow indicates protein tubulin; red arrow indicates small amount of interstitial inflammatory cell infiltration; black arrow indicates renal tubular atrophy. The pale green arrow indicates collagen deposition in the glomerular basement membrane; the pale green arrow indicates renal interstitial fibrosis). Model group vs. control group (*p < 0.05, **p < 0.01, ANOVA followed by LSD); ZWD and prednisone groups vs. model group (#*p <* 0.05, ##*p <* 0.01, ANOVA followed by LSD). ZWD: Zhenwu Decoction; LSD: least significant difference; ANOVA: Analysis of Variance.

Renal histopathological changes and collagen deposition were observed using H&E and Masson’s trichrome staining. In the control group, the kidneys appeared normal and dark red, whereas those in the ADR group were ischemic and yellow (Figure 4(A)). ZWD improved ischemia in NS rats. H&E staining revealed clear and uniform structures for the renal glomeruli, tubules, and interstitium in the control group, whereas the ADR group displayed renal tubular atrophy and dilation, protein casting, and infiltration of inflammatory cells (neutrophils and lymphocytes, Figure 4(B)). NS rats treated with ZWD or prednisone showed improvement (*p* < 0.05, Figure 4(D)). Masson’s trichrome staining revealed that the glomerular basement membrane was intact and the mesangial matrix was evenly distributed in the control group (Figure 4(C)), whereas the ADR group exhibited collagen deposition and interstitial fibrosis (*p* < 0.01, Figure 4(E)). The ZWD and prednisone treatments alleviated collagen deposition in the glomerular basement membrane and reduced interstitial fibrosis associated with NS (*p* < 0.01).

### 3.5. ZWD ameliorates renal function in NS

**Figure 5.**
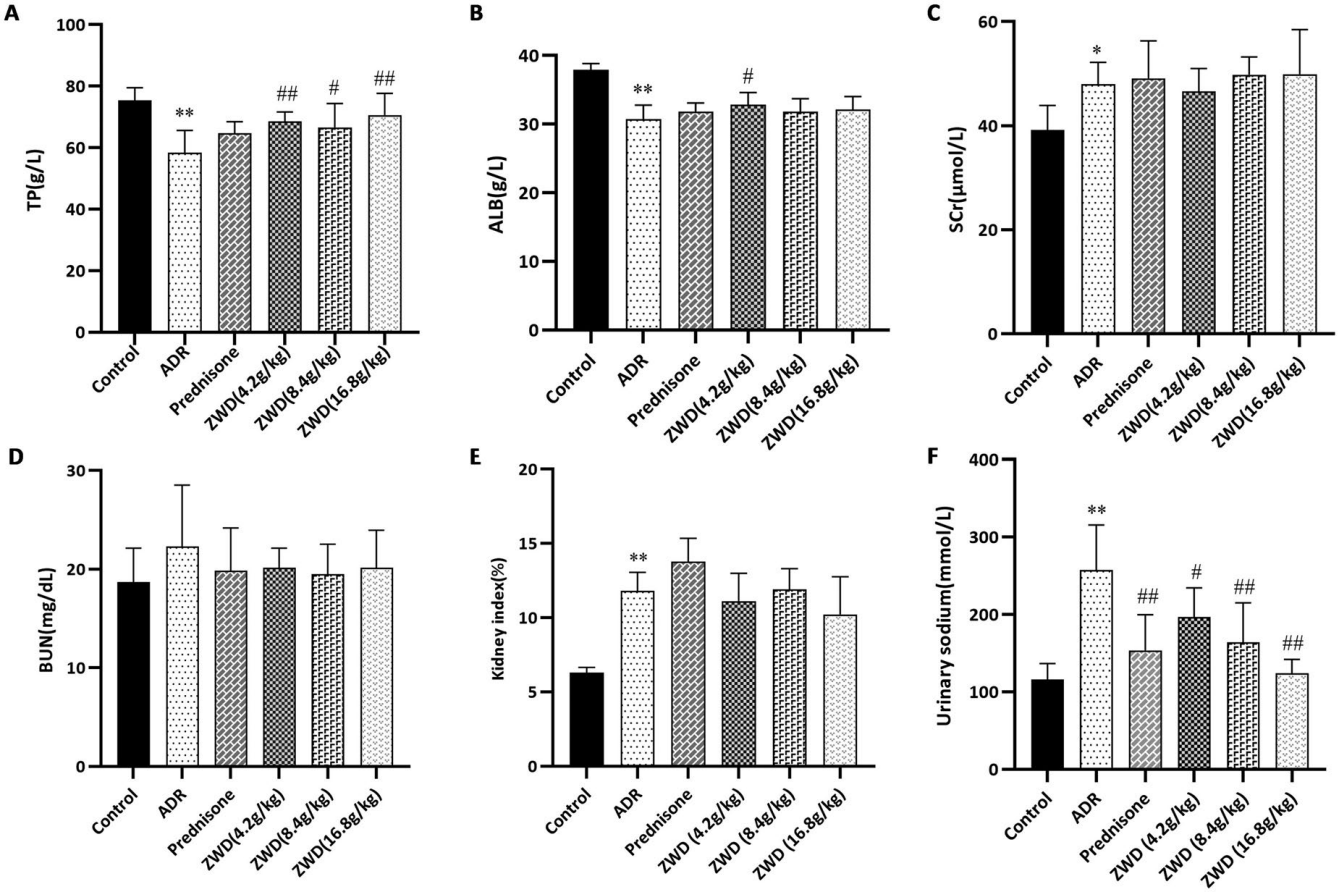
ZWD improves renal function in NS. (A) Serum TP (*n* = 6). (B) ALB (*n* = 6). (C) Scr (*n* = 6). (D) BUN (*n* = 6). (E) Kidney index (*n* = 6). (F) Urinary sodium (*n* = 6). Model group vs. control group (*p < 0.05, **p < 0.01, ANOVA followed by LSD); ZWD and prednisone groups vs. model group (^#^*p <* 0.05, ^##^*p <* 0.01, ANOVA followed by LSD). ZWD: Zhenwu Decoction; LSD: least significant difference; BUN: blood urea nitrogen; ANOVA: Analysis of variance.

After injection with ADR for 6 weeks, the serum Scr, urine sodium, and the kidney index increased and serum TP and ALB levels decreased compared with those in the control group (*p* < 0.05, Figure 5(A–C,E)). The serum BUN level increased in the ADR group, though not significantly so (Figure 5(D)). Compared to the ADR group, ZWD significantly enhanced serum TP and ALB levels and lowered urine sodium levels (*p* < 0.05), but had no significant effect on serum Scr, BUN, or the kidney index. The Prednisone group significantly reduced urine sodium levels only (*p* < 0.01), without significantly affecting other indicators. These findings showed that ZWD ameliorated hypoproteinemia and sodium loss in NS and it exerts more potent therapeutic effects than prednisone.

### 3.6. ZWD improves the renal water and sodium disturbances in NS

**Figure 6.**
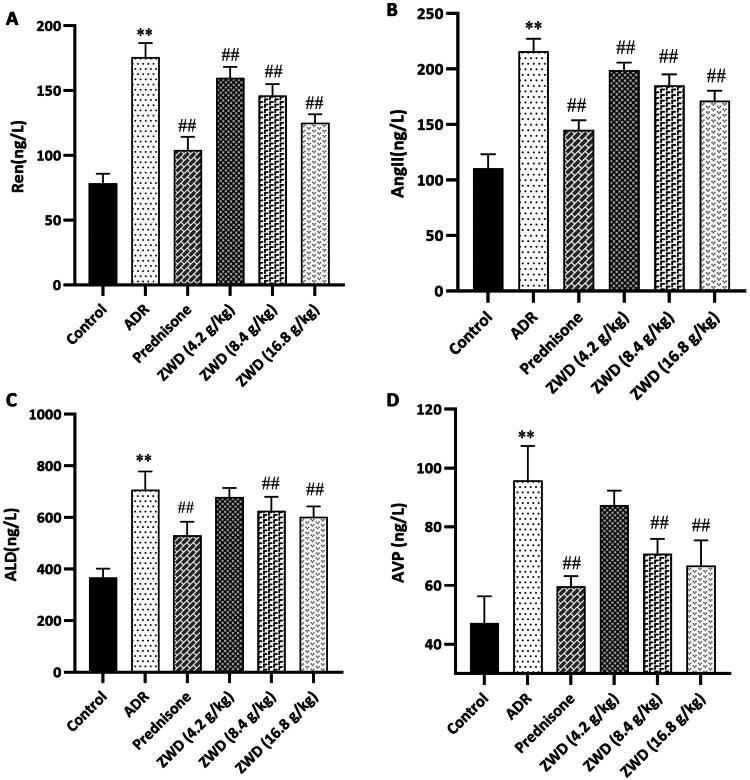
ZWD improves renal water and sodium imbalance in NS. (A) Serum Ren (*n* = 6). (B) AngII (*n* = 6). (C) ALD (*n* = 6). (D) AVP. (*n* = 6). Model group vs. control group (**P < 0.01, ANOVA followed by LSD); ZWD and prednisone groups vs. model group (^##^*P <* 0.01, ANOVA followed by LSD). AVP: arginine vasopressin; ALD: aldosterone.

**Figure 7.**
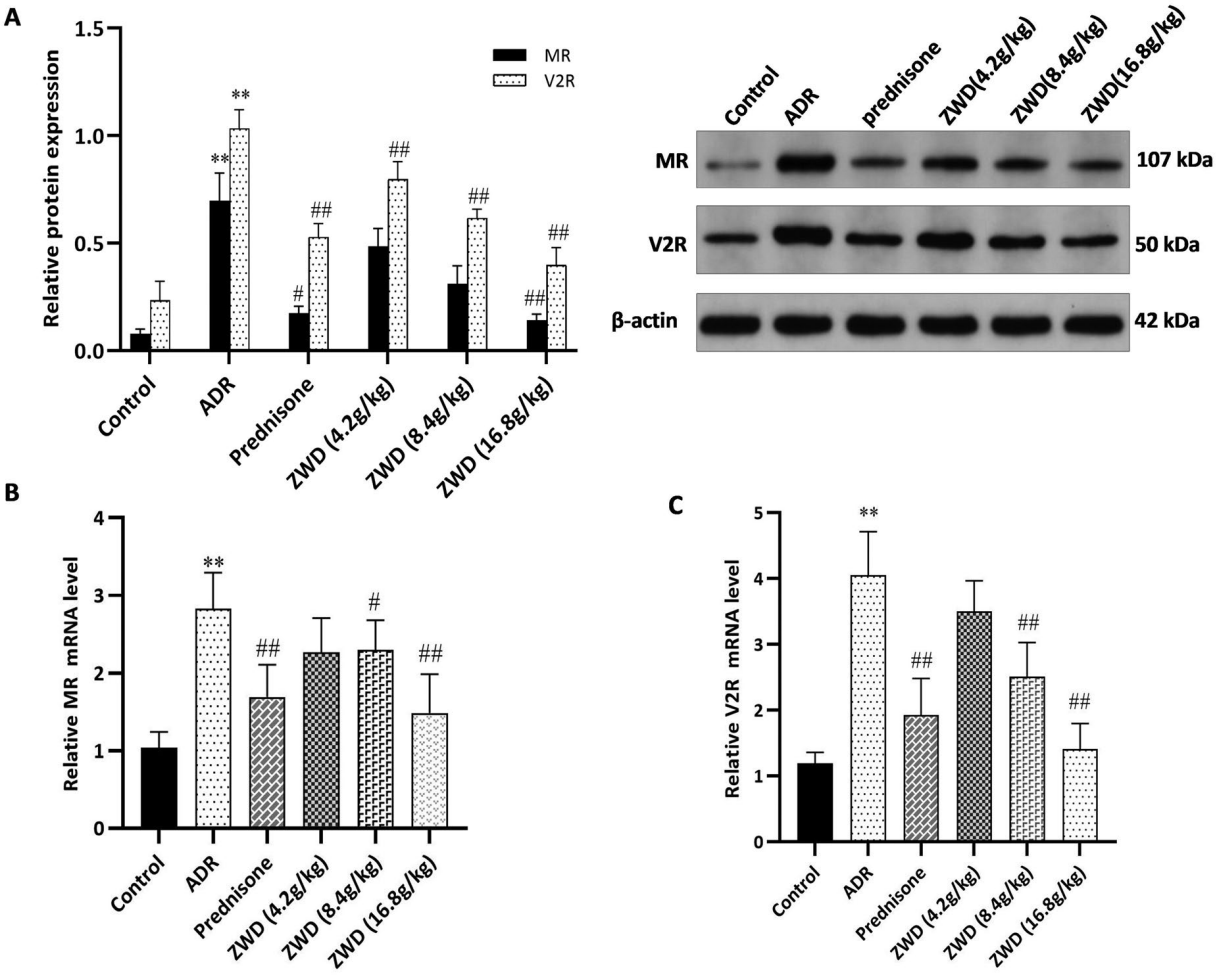
ZWD inhibits renal MR and V2R expression in NS. (A) Protein levels of MR and V2R detected by WB (*n* = 6). (B–C) mRNA levels of MR and V2R detected by RT-qPCR (*n* = 6). Model group vs. control group (**p < 0.01, ANOVA followed by LSD); ZWD and prednisone groups vs. model group (#*p <* 0.05, ##*p <* 0.01, ANOVA followed by LSD). V2R: type 2 vasopressin receptor; MR: mineralocorticoid receptor; ZWD: Zhenwu Decoction; LSD: least significant difference; WB: western blot.

ADR-induced NS rats showed a remarkable decrease in 24 h urine excretion compared to control rats, indicating some water retention associated with NS (Figure 2(B)). We assessed whether ZWD ameliorated renal water and sodium imbalance based on the serum levels of Ren, AngII, ALD, and AVP. Compared to the control group, the serum levels of Ren, AngII, ALD, and AVP increased in the ADR group (*p* < 0.05), and these changes were reversed by ZWD administration (*p* < 0.01, Figure 6(A–D)). We assessed the downstream receptor levels of RAAS and AVP in the kidneys using WB and RT-qPCR. WB analysis showed that MR and V2R levels were higher in the ADR group than in the control group (*p* < 0.01), and these changes were reversed by ZWD (*p* < 0.05, Figure 7(A)). The RT-qPCR analysis demonstrated consistent trends in mRNA levels (*p* < 0.05, Figure 7(B,C)). These results indicate that ZWD suppressed the RAAS system and release of AVP to ameliorate renal water and sodium imbalance in NS rats.

### 3.7. ZWD attenuates water retention by suppressing renal AQP levels in NS

**Figure 8.**
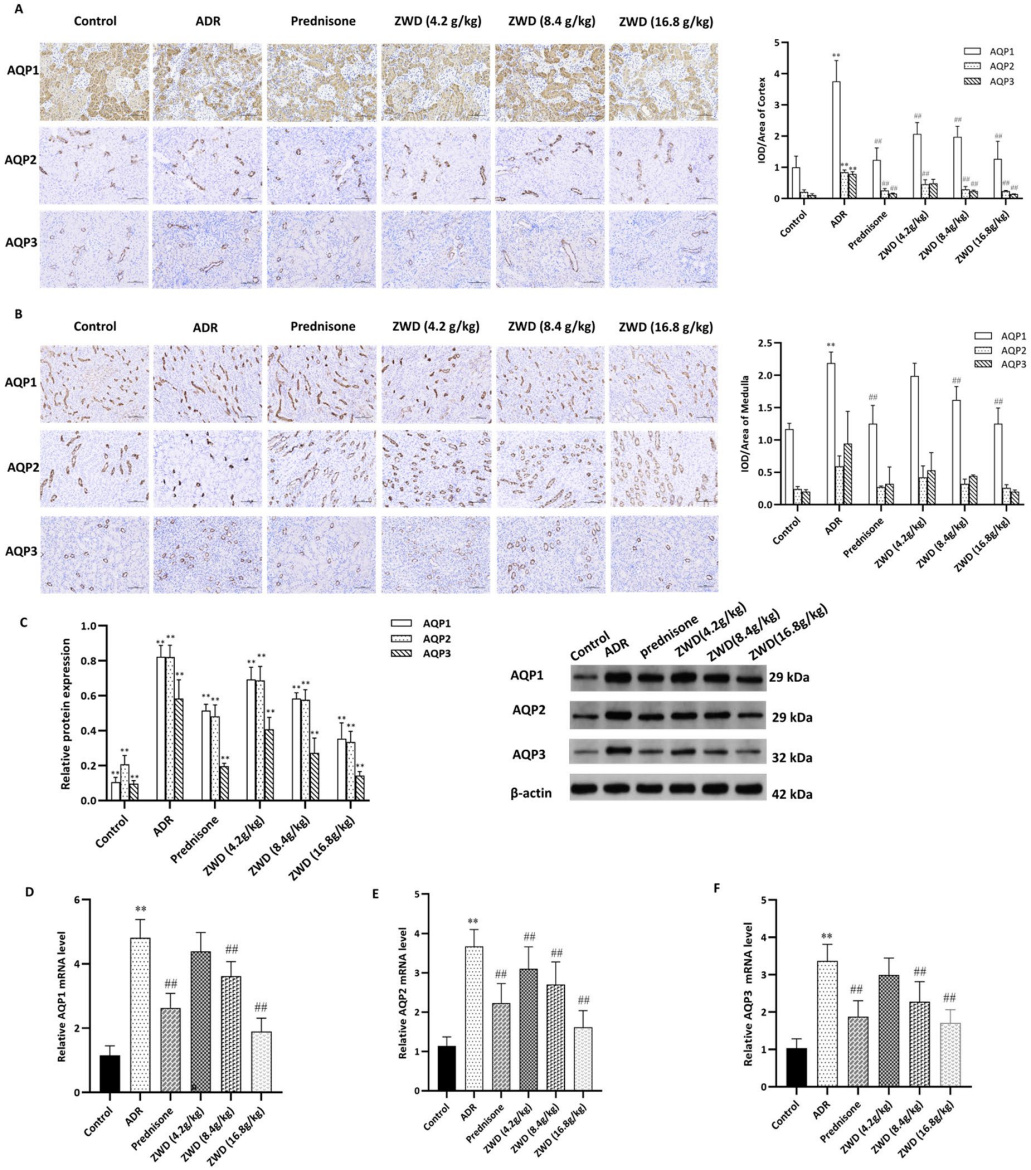
ZWD attenuates renal water reabsorption *via* suppressing AQPs in NS. (A-B) Location of AQP1, AQP2, and AQP3 in cortex (A) and medulla (B), detected by IHC. (C) Protein levels of AQP1, AQP2, and AQP3, detected by WB (*n* = 6). (D-F) mRNA levels of AQP1, AQP2, and AQP3 detected by RT-qPCR (*n* = 6). Model group vs. control group (**p < 0.01, ANOVA followed by LSD); ZWD and prednisone groups vs. model group (#*p <* 0.05, ##*p <* 0.01, ANOVA followed by LSD). AQP: aquaporin; WB: western blot; ANOVA: analysis of variance.

AQPs are highly expressed in the kidneys and play critical roles in water reabsorption. Overexpression of AQP2 can result in water balance disorders including NS [2]. IHC, WB, and RT-qPCR were performed to determine whether ZWD improves water and sodium imbalances by regulating renal AQP expression. IHC analysis (Figure 8(A,B)) showed that compared to the control group, the ADR group had significantly elevated expression of AQP1, AQP2, and AQP3 (*p* < 0.01) in the renal cortex, but only AQP1 (*p* < 0.01) in the medulla. Compared with the ADR group, the ZWD group exhibited significantly decreased expression of AQP1, AQP2, and AQP3 in the renal cortex and of AQP1 in the medulla (*p* < 0.01). However, the expression of AQP2 and AQP3 in the medulla showed a decreasing trend, but the change was not statistically significant. WB results (Figure 8(C)) showed similar trend to the IHC findings. The protein expression of AQP1, AQP2, and AQP3 in the kidney was significantly higher in the ADR group than in the control group (*p* < 0.01). Oral administration of ZWD downregulated the protein expression of AQP1, AQP2, and AQP3 in NS rats (*p* < 0.01). The RT-qPCR analysis showed consistent trends in terms of the mRNA levels (*p* < 0.01, Figure 8(D–F)). The therapeutic outcomes of prednisone were equivalent to those of ZWD. These results indicate that ZWD exerts potent diuretic effects by attenuating renal water reabsorption mediated by AQP1, AQP2, and AQP3 in NS. WB results (Figure 8(C)) showed similar trend to the IHC findings.

### 3.8. Compound-major target molecular docking

**Figure 9.**
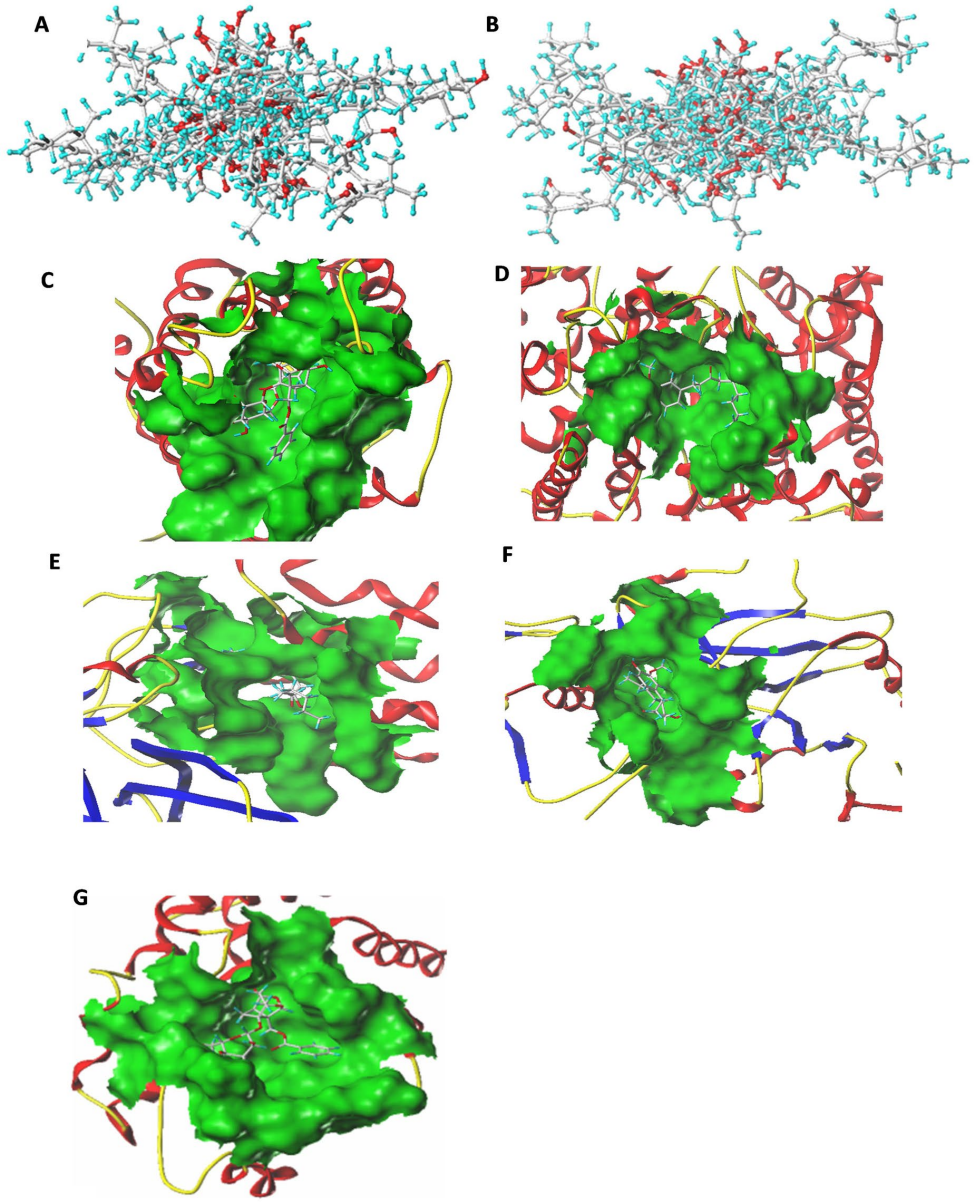
Compound–major target molecular docking analysis. (A) Initial 3D conformation of unoptimized bioactive compounds. (B) 3D conformation of optimized bioactive compounds. (C) Interaction mode of AQP1 with benzoylpaeoniflorin. (D–F) Interaction mode of AQP2, V2R, and MR with 6-gingerol. (G) Interaction mode of AQP3 with paeoniflorin. AQP: aquaporin; V2R: type 2 vasopressin receptor; MR: mineralocorticoid receptor.

The active components of ZWD were structurally optimized using SYBYL-X 2.1.1. Figure 9(A) displays the initial 3D conformation of the unoptimized bioactive compounds. Energy minimization employing the Tripos force field was performed on small-molecule ligands to obtain geometrically refined structures and reasonable molecular conformations (Figure 9(B)). The molecular docking analysis (Table 2) identified 13 compounds with significant binding affinity for AQP1 (T_Score > 5), with benzoylpaeoniflorin demonstrating the strongest interaction (T_Score > 7; binding pocket in Figure 9(C)). Eleven compounds showed substantial affinity for AQP2 (T_Score > 5), and 6-gingerol achieved optimal binding (T_Score > 8; Figure 9(D)). Six compounds effectively bound to V2R (T_Score > 5), with 6-gingerol exhibiting maximal affinity (T_Score > 6; Figure 9(E)). Similarly, six compounds exhibited significant binding to MR (T_Score > 5) with 6-gingerol showing the highest binding potential (T_Score > 8; Figure 9(F)). Eight compounds exhibited significant binding with AQP3 (T_Score > 5), with paeoniflorin displaying the highest binding potential (T_Score > 8; Figure 9(G)).

**Table 2.** Molecular docking analysis of ZWD bioactive compounds (T_score).

NO.	Chemical name	AQP1	AQP2	AQP3	AVP/V2R	MR
1	Karakoline	3.4647	3.792	4.3779	3.4807	1.978
2	(S)-Norcoclaurine	4.1719	5.6563	3.4040	4.9038	5.813
3	Benzoylmesaconine	5.745	4.5391	6.2607	3.6257	0.4453
4	Benzoylhypaconine	5.6984	4.0786	3.1420	2.3459	4.645
5	Benzoylaconine	6.0764	4.2724	4.4789	1.4574	1.4114
6	Paeoniflorin	6.0336	7.2393	6.8253	6.7852	2.7072
7	Albiflorin	6.6897	6.907	5.5026	5.7099	5.9562
8	Lactiflorin	5.4823	5.6497	6.2527	3.355	4.755
9	Benzoylpaeoniflorin	7.9935	6.2212	5.3991	5.302	5.3652
10	Pachymic acid	6.4793	5.3453	5.8201	4.3977	5.3341
11	Poricoic acid A	6.7318	6.6296	3.3901	5.1892	3.7942
12	Poricoic acid C	6.3886	6.0606	2.9182	3.5488	3.8478
13	Tumulosic acid	6.2605	5.6752	5.5153	3.6404	4.919
14	6-Gingerol	6.5635	8.5244	5.9711	6.860	8.2689
15	Atractylenolide I	3.8426	3.3106	2.9219	3.4398	4.1397
16	Atractylenolide III	4.0376	4.142	3.3795	6.7852	3.1223
17	3β-Hydroxyatractylon	4.0376	4.142	3.3795	3.8913	3.1223
18	(+)-Catechin	5.5347	6.1265	3.5069	5.7162	5.4281

## 4. Discussion

NS is a common clinical syndrome characterized by damage to podocytes that results in significant proteinuria, edema, hyperlipidemia [19,20]. The pathological process is generally exacerbated *via* the activation of the RAAS and AVP pathways [21] and develops into water and electrolyte disorders. Animal models of adriamycin-induced nephropathy are commonly used in modern research on NS [22,23]. In the present study, the ADR-induced NS model exhibited severe proteinuria compared with the control group, confirming its successful construction. Administration of ZWD significantly reduced proteinuria, reversed body weight loss, and increased urine excretion in NS rats, consistent with previous findings [12].

The effect of ZWD on serum lipid levels in NS rats was assessed in this study. Although ZWD did not exert a significant inhibitory effect on serum TG, TC, and HDL-C levels, serum LDL-C levels decreased in a dose-dependent manner. LDL-C upregulation is an independent risk factor for the progression of kidney disease, and reducing hyperlipidemia may improve kidney prognosis [24]. These results suggested that ZWD regulates serum lipid levels *in vivo* and are largely consistent with previous findings in animal experiments with ZWD [10,16].

The reversal effect of ZWD on kidney injury was subsequently examined. ALB, the primary component of TP, is a major modulator of fluid distribution in different parts of the body since it strongly determines a large proportion of the plasma colloid osmotic pressure[25]. ALB may be a core target of ZWD in the treatment of heart failure [26]. Our results demonstrated that ZWD was more effective in increasing the levels of serum TP and ALB than prednisone, indicating that ZWD could effectively improve hypoproteinemia in NS, and thus help reduce proteinuria, which is similar to the effects of tripterygium glycosides in the intervention in NS rats [17]. The detection of kidney index, the serum BUN and Scr levels, and urine sodium level can reflect the severity of kidney injury [17,27]. Our findings showed that ZWD ameliorated sodium loss in NS, suggesting its therapeutic potential for reversing renal injury. In this study, the results of H&E and Masson’s trichrome staining indicate that ZWD could effectively reduce renal protein casting and infiltration of inflammatory cells and fibrosis in NS, thus reducing proteinuria. Our histopathological findings were consistent with those of Liang et al. [9], suggesting similar potential mechanisms for the anti-inflammatory actions of ZWD [28]. In addition, the significant attenuation of renal fibrosis by ZWD not only underscores its potential for treating nephrotic syndrome but also suggests its utility in targeting fibrotic processes common to chronic kidney disease progression [29].

RAAS plays a crucial role in regulating body fluid homeostasis and maintaining water and electrolyte balance [30]. The major hormones in the RAAS—AngII can regulate AVP-induced water reabsorption by stimulating the secretion of AVP [31,32], which in turn determines urine concentration *via* the regulation of water permeability of the renal CD. In our study, 24 h urine excretion decreased markedly in NS rats compared to control rats, indicating some water retention, consistent with established findings [12]. ZWD greatly downregulated the serum levels of Ren, AngII, ALD, and AVP, and the protein and mRNA expression of the corresponding receptors MR and V2R in the kidney tissue of NS rats, suggesting that ZWD may suppress the RAAS system and release of AVP to ameliorate renal water and sodium imbalances in NS. These findings align with the underfill hypothesis, which proposes that NS-induced edema could be exacerbated by the activation of RAAS and release of AVP [21], and are consistent with prior research on the protective effect of ZWD on renal injury [33]. Our findings integrate the molecular understanding of RAAS/AVP signaling in nephrology with the holistic intervention strategy of Traditional Chinese Medicine (TCM), demonstrating a convergent therapeutic paradigm.

After revealing the therapeutic outcomes of ZWD in NS rats, we investigated the specific molecular mechanisms. AQPs are a family of membrane proteins that function as water channels and play crucial roles in renal water reabsorption [34]. Key members of this family (such as AQP1, AQP2, etc.) are crucial contributors to renal physiology and fibrotic pathology *via* multiple mechanisms, encompassing imbalance of water transport, epithelial-mesenchymal cell transformation and inflammatory response [35]. AQP1 is an essential water-specific channel that mainly occurs in the plasma membrane of the proximal convoluted tubules and participates in water homeostasis. AQP2 is distributed on the apical membranes of the CDs and ultimately modulates water reabsorption *via* the AVP-V2R pathway. AVP-modulated AQP2 transfer and expression is enhanced by AngII [31]. AQP2 overexpression can result in water imbalances in humans with NS [2]. AQP3 is distributed on the basolateral plasma membrane of CDs and regulates water reabsorption *via* AQP2, and is in turn modulated by ALD [6]. The current IHC results showed that the renal distributions of AQP1, AQP2, and AQP3 were the same as previously described [36]. The protein and mRNA levels of AQP1, AQP2, and AQP3 in the ADR group were significantly higher than those in the control group, consistent with previous findings [2,3]. WB and RT-qPCR showed that the protein and mRNA levels of AQP1, AQP2, and AQP3 in the ADR group were significantly higher than those in the control group, consistent with previous findings [3,15]. However, some studies demonstrated that renal AQP2 expression was significantly lower in NS rats than in normal rats [12]. This seemingly contradictory result may be due to differences in the dose and duration of ADR administration. Treatment with ZWD for 4 weeks significantly downregulated the renal protein and mRNA levels of AQP1, AQP2, and AQP3 in a dose-dependent manner, consistent with the increased urine excretion and decreased serum RAAS and AVP levels and recent findings [37]. Nevertheless, some studies showed that ZWD increased renal V2R and AQP2 expression to improve edema in NS rats [12,38]. Therefore, the contradictory results may also be due to differences in the types of cells or tissues. Overall, our results suggest that ZWD may exert its diuretic effect by attenuating renal water reabsorption *via* AQP1, AQP2, and AQP3 in NS, and AQPs are central mediators of NS edema. Thus, elucidating their mechanisms to develop targeted interventions holds promise for the precise treatment of renal diseases.

Molecular docking analysis revealed that the three active components of ZWD (benzoylpaeoniflorin, 6-gingerol, and paeoniflorin) exerted synergistic effects on NS-induced edema markers (MR, V2R, and AQP1-3). In particular, 6-gingerol demonstrated optimal binding affinity for MR, V2R, and AQP2. Previous research showed that 6-gingerol treatment attenuates renal damage by regulating oxidative stress and inflammation, which further validates our results [39]. 6-Gingerol may exert a greater influence than other constituents on NS-induced edema. Notably, favorable binding between pachymic acid and AQP1, AQP2, and AQP3 (T_Score > 5), consistent with established findings [14,40], suggests a role in regulating water permeability in NS. Benzoylpaeoniflorin and paeoniflorin showed optimal binding affinities for AQP1 and AQP3, respectively. A previous study showed that paeoniflorin exerted anti-AngII effects by modulating oxidative stress and the Nrf2 signaling pathway [41], consistent with the effect of ZWD on RAAS downregulation in our study. This synergistic interaction between multiple components underscores the value of investigating TCM through the framework of modern natural product pharmacology [42], offering a systems-level strategy for complex diseases like NS.

Furthermore, ZWD (in our NS model) demonstrated efficacy comparable to prednisone [11], but through a distinct, multi-targeted mechanism. Unlike the singular focus of prednisone (immunosuppression) or RAAS inhibitors (blocking a single pathway), ZWD coordinated the amelioration of multiple pathological processes in NS, including impaired water balance, hypoproteinemia, hyperlipidemia, and fibrosis. This integrated effect may be attributed to its concurrent modulation of pivotal targets, such as AQP signaling pathways [35]. The multi-targeted nature of ZWD may help circumvent common limitations of mono-therapies, particularly glucocorticoid resistance. Thus, ZWD exemplifies a holistic, systems-level therapeutic strategy rooted in natural product pharmacology.

Although this study presents evidence on the therapeutic potential of ZWD in NS, it has certain limitations. While therapeutic efficacy manifested predominantly in later disease stages, the dose-response trajectories imply continuous severity modulation rather than exclusive endpoint modification. In addition, emerging evidence implicates epithelial sodium channel (ENaC) dysregulation in nephrotic sodium retention[3]. Future studies should examine sodium handling pathways, including ENaC, NHE3, and NCC, to fully elucidate anti-edema mechanism of ZWD. Finally, the treatment effects of ZWD combined with prednisone or other angiotensin converting enzyme inhibitors were not assessed in this study and should be investigated in the future.

## 5. Conclusions

This study demonstrated that ZWD can ameliorate proteinuria, pathological damage, and fibrosis in renal tissue, and reduce water retention by downregulating the AVP-VR2-AQP2 and RAAS-MR-AQP3 pathways to intervene in ADR-induced NS in rats. Molecular docking analysis indicated that 6-gingerol and paeoniflorin bound best to proteins of the V2R-AQP2 and MR-AQP3 pathways. Further monomeric drug studies are necessary to explore the specific pharmacological effects of ZWD and promote its clinical application against NS. Overall, our findings suggest a novel therapeutic strategy that may be applicable to a wider spectrum of kidney diseases characterized by water retention and fibrosis, beyond NS.

## Data Availability

The data supporting the findings of this study are available upon request from the corresponding author.
